# Psychosocial and Behavioral Effects of the COVID-19 Pandemic in the Indian Population: Protocol for a Cross-sectional Study

**DOI:** 10.2196/29896

**Published:** 2021-11-05

**Authors:** Megha Joshi, Aangi Shah, Bhavi Trivedi, Jaahnavee Trivedi, Viral Patel, Devam Parghi, Manini Thakkar, Kanan Barot, Vivek Jadawala

**Affiliations:** 1 Department of Psychiatry Shrimati Nathiba Hargovandas Lakhamichand Municipal Medical College Gujarat University Ahmedabad India; 2 Boston Children's Hospital Harvard University Boston, MA United States; 3 Byramjee Jeejabhoy Medical College Gujarat University Ahmedabad India; 4 Department of Internal Medicine University of Louisville Louisville, KY United States; 5 Department of Internal Medicine Texas Tech University Health Sciences Center El Paso El Paso, TX United States; 6 Department of Internal Medicine Landmark Medical Center New York Medical College Woonsocket, RI United States; 7 Medical College Baroda Aanandpura City - Vadodara India; 8 Department of Internal Medicine Southeast Health Dothan, AL United States; 9 Department of Psychiatry Brandywine Hospital Tower Health Coatesville, PA United States; 10 Department of Orthopaedics Acharya Vinoba Bhave Rural Hospital Datta Meghe Institute of Medical Sciences Wardha India

**Keywords:** COVID-19, mental health, India, lockdown, isolation, social isolation, behavior, psychology, psychosocial effects

## Abstract

**Background:**

During the year 2020, the COVID-19 pandemic spread from China to the rest of the world, which prompted the world to implement a widespread mandated quarantine or social isolation. The impending uncertainty of the pandemic must have resulted in a variety of widespread mental health maladies. There has been documentation in the literature about a lot of these in small populations of the world but limited studies have been conducted in India, leading to limited evidence in the literature.

**Objective:**

The main objective of our study is to investigate the mental health effects that the COVID-19 pandemic has had on the general population in India both quantitatively and qualitatively. These results will help contribute to reducing the knowledge gap that is recognized in the literature, which is the result of the unprecedented and novel nature of the pandemic.

**Methods:**

We designed and validated our own questionnaire and used the method of circulating the questionnaire via WhatsApp (Facebook Inc). WhatsApp is a social media app that is very popularly used in India; hence, it turned out to be an effective medium for gathering pilot data. We analyzed the pilot data and used them to validate the questionnaire. This was done with the expertise of our mentor, Nilima Shah, MD (psychiatry). We gathered pilot data on 545 subjects and used the results to determine the changes that were needed for the questionnaire while simultaneously validating the questionnaire.

**Results:**

The study protocol was approved in September 2020 by the institutional review board at Vadilal Sarabhai General Hospital, Ahmedabad, Gujarat, India.

**Conclusions:**

The following preliminary assumptions can be made about the study based on the pilot data: the majority of the survey respondents were male (289/545, 53%), most of them were educated and employed as health care workers (199/545, 36.5%). The majority of the responders were self-employed (185/545, 33.9%), single (297/545, 54.5%), and stayed with their families (427/541, 79%) for the lockdown, which helped them psychologically. Findings that are specific to mental health have been elaborated upon in the manuscript. It is evident from the data collected in previous literature that the pandemic has had significant detrimental effects on the mental health of a vast proportion of the Indian population.

**International Registered Report Identifier (IRRID):**

DERR1-10.2196/29896

## Introduction

### Background

From March 2020 to June 2020, most of the world underwent social isolation, mandated quarantine, or lockdown to prevent the excessive spread of and further casualties from COVID-19. Such isolation has caused a dramatic change in routine livelihoods. Although the isolation was essential to containing the disease’s spread, we argue that this drastic change in daily life must have led to several psychological issues that are mainly the result of the uncertain nature of the disease; hopelessness about the future; a lack of motivation due to the existential crisis posed by the disease; occupational and financial difficulties; and several novel, day-to-day struggles involving food and family [1-21].

Our preliminary review of the literature suggests that there has been a substantial increase in the incidence of mental health disturbances in people, including symptoms within the full spectrum of anxiety disorders, depression, acute stress disorder, posttraumatic stress disorder, and alcohol and substance use disorders. Numerous other studies have reported an increase in the incidence of deteriorating work performance; insomnia; and feelings of fear, apprehension, helplessness, confusion, anger, and frustration among the general population and frontline health care workers, and this has been associated with the COVID-19 lockdown [1-5,7]. There is documentation of the increased incidence of depression symptoms in young and single individuals. Stressful jobs, exposure to COVID-19 and the risk of such exposure in the workspace, and forced lockdowns during the outbreak are some of the major factors that are associated with the increased reporting of psychological disturbances [3]. The COVID-19 lockdown and the resulting psychological impact could also have resulted in multiple changes in drinking habits. We found an increase in the consumption and purchase (both in-store and web-based purchases) of alcohol, and this correlates with the increase in the duration of the lockdown. Individuals with a lower level of education and those with higher levels of perceived stress have been identified to be at the highest risk for such behavior [8]. There have also been findings of improved social support among friends and families during the lockdown and increased attention to mental health due to more time being allocated to relaxation during the lockdown [7].

Given the evidence found in literature and the unique nature of the COVID-19 pandemic, which has had unique and varied effects on the general population at the individual level and as a whole (ie, effects that are not in line with those of any particular or established clinical syndromes), we want to conduct a cross-sectional survey of the Indian population with an innovative, validated questionnaire to assess the mental health of and psychosocial changes in people who have been affected by the pandemic. Similar studies are being conducted in various other regions of the world as well [22].

There is a lack of evidence and research in the literature about the mental health of the Indian population [20]. This has resulted in a lack of awareness of the symptoms and presentations of different mental health disorders in the general public [20]. Therefore, we chose this target population to help us recognize the culture-specific and general effects that the COVID-19 lockdown and social isolation have had on their psyche. The data from this study can provide valuable and much sought-after insight into the mental status of the general public in the South Asian (Indian) population, which would be useful to mental health professionals and public health experts when making informed decisions that would eventually benefit the general public. Importantly, these interventions need to be based on the community level, since this is the population level that the pandemic has affected the most [23]. These changes need to be similarly reflected at the systemic level in order to maximize their benefit.

### Statement of Purpose

Our study has qualitative and quantitative arms. The qualitative arm of this study aims to analyze the psychosocial and behavioral effects of social isolation and mandated quarantine or lockdown. This arm includes an assessment of the Indian population’s awareness of and knowledge about COVID-19. The quantitative arm of the study aims to examine the extent of the association between psychosocial and behavioral effects and the various demographic factors of the target population (eg, age, sex, etc). The main purpose of our study is to determine the present mental health status of the general population and the direct effects of social isolation resulting from the COVID-19 pandemic.

### Hypotheses and Aims

Hypothesis 1.1 is as follows: there are multiple adverse psychosocial and behavioral effects among remote workers, students, and students transitioning to the workforce that have arisen because of the COVID-19 pandemic lockdown.

Aim 1.1 is as follows: we aim to qualitatively analyze the psychosocial and behavioral effects among the target population via web-based survey forms and present these forms in an easily readable manner.

Hypothesis 1.2 is as follows: the multiple psychosocial and behavioral effects are associated with and vary due to the demographic factors of the target population, such as age, sex, education level, the area of education, ethnicity, the location of residence, relationships, and employment status.

Aim 1.2 is as follows: we aim to quantitatively analyze and determine the extent of these associations and their statistical significance.

### Objectives

Aim 1.1 will be accomplished by collecting data via the use of a Google Forms (Google LLC) survey and by using Google Forms software to present data in the form of different kinds of charts, such as bar and pie charts. These will be included in the *Tables and Charts* section of the poststudy research article. Aim 1.2 will be accomplished by using Stata software (StataCorp LLC) to analyze the demographic variables across the data on psychosocial and behavioral effects via methods such as logistic regression and chi-square analysis. The methods used will depend on the kinds of variables being analyzed. The results of this analysis will also be included in the *Analysis* section of the poststudy research article.

## Methods

### Procedures

We used, and want to continue using, the snowball sampling method. We digitized our validated questionnaire via the use of Google Forms software. Afterward, we contacted everyone we knew and sent them links to the Google Forms questionnaire via different social media platforms and apps, such as WhatsApp (Facebook Inc), Facebook Messenger (Facebook Inc), SMS text messaging, and email. We made sure to divide our contacts before sending out the survey to avoid the duplicity of data, but there is a certain margin of error that is to be expected with this method of data collection. This is a limitation of our data collection method. Further, since Google Forms does not record IP addresses, there is no way of knowing who filled out the forms. There is another method that can be used; while surveying a target population, we can restrict access to Google Forms and send the questionnaire to members of a particular group. We attempted to mitigate the error of duplicity by turning on the “limit to one response” option in the Google Forms survey settings. This will require survey responders to sign in with their Gmail or Google accounts before they can respond to the survey. This can potentially compromise the blinding of the subjects; however, we can delete respondents’ personal information on the Google Forms platform to protect privacy and maintain confidentiality. This will help with gathering more meaningful data. We want to continue gathering data to create a large data set with satisfactory power and the required effect size for conducting statistical analyses.

### Setting and Sample

The study is ongoing, and so far, 500 adult and pediatric individuals (age group: range 15-70 years) have responded to the questionnaire. We used a cross-sectional study design that involved the use of the snowball sampling method. We distributed a questionnaire to known friends, family members, and colleagues and asked them to pass it on to their acquaintances. Due to the worldwide pandemic and the need to reduce the amount of physical interactions with human subjects, we developed a web-based questionnaire by using Google Forms. To date, the preliminary data of 500 subjects have been collected. These data were collected over a period of 40 days (since October 6, 2020). We want to gather more data once and if the institutional review board grants their approval.

### Inclusion and Exclusion Criteria

The inclusion criteria are as follows: respondents aged >15 years (as approved by the institutional review board) and people who filled out the entire survey. The exclusion criteria are as follows: respondents aged ≤15 years and people who did not fill out the entire survey.

### Survey Development

The questionnaire was formed with the help of Google Forms software. The questions are based on the templates of the *Diagnostic and Statistical Manual of Mental Disorders, 5th Edition* (*DSM-5*) questionnaire for depression and anxiety; the Patient Health Questionnaire-9; and the Primary Care Evaluation of Mental Disorders diagnostic questionnaire. We used the ideas that formed the basis of the questions in these questionnaires to address our specific research questions and avoid gathering unnecessary data. This method of using ideas from standard forms has been used previously and has been proven to be effective in answering the desired research questions [6-9]. We did not want to use standard scales as the answer format because they are usually harder to interpret and can make the response process difficult. This is why we used simplified versions of answer options in our questionnaire.

Questions 1 to 4 have been used to collect basic demographic information, such as race, age, and the area of residence. Associations between particular demographic factors and psychosocial symptoms have previously been demonstrated in the literature, so we want to take these factors into consideration in the final data analysis as well [10].

Questions 5 to 7 have been used to collect information regarding qualifications, the field of work, and employment status. The rationale behind these questions is that due to recent unforeseen conditions, many people have lost their jobs or have been rendered unemployed before they could start new jobs. This has directly and indirectly affected their mental health, as there has been an increase in the incidence of mental health disorders, including anxiety and depression, resulting from feelings such as the loss of control, hopelessness, the hyperawareness of economic losses [11,12].

Questions 6 to 8 pertain to whom respondents spent the lockdown period with and their social interactions with relatives, friends, and other people. These questions enable us to better understand how these important interpersonal relationships impact respondents’ mental well-being [13].

With questions 9 to 14, we have tried to capture data on the changing sleep and eating habits, physical activities, and hobbies of individuals. These factors are generally reflective of the mental status of individuals, as evidenced in literature and by the *DSM-5* questionnaire [14,16,17].

Questions 22 to 24 ask about whether individuals or any loved ones are infected with SARS-CoV-2 and the availability of adequate testing and treatment options in their vicinity. The knowledge of infections occurring anywhere near a person may cause a state of paranoia, worry, and anxiety [2].

Questions 28 to 29 are about substance abuse during the lockdown periods. There have been numerous studies that indicate how social isolation affects the already present habits of individuals and results in the development of newer habits of substance abuse as a maladaptive method of coping [4,8,16]. The rest of the questions—questions 16 to 21, 25 to 27, and 30—are about self-reported psychomotor symptoms of depression and anxiety from the *DSM-5* criteria [16,17].

We validated the questionnaire with the help of available pilot data. We first established face validity via consultation with Indian experts in the field. Afterward, we cleaned the collected data by using principal component analysis and calculated the Cronbach α to establish the internal consistency of the questionnaire. These methods have been used numerous times before to establish the validity of an innovative questionnaire [18]. A statistician assisted us with these techniques.

### Data Analysis

We will be using Stata software to calculate power and effect size and to analyze the trends in mental disorder symptoms, sleeping and eating habits, physical activity, remote and in-person social interactions, and the mental state of sampled individuals during the period of social isolation. We will be using a mixed methods analysis in the study, that is, the data will be analyzed both qualitatively and quantitatively. The data will be used for the qualitative portion of the analysis, and they will be converted into scores and used as continuous, ordinal, or categorical variables for the quantitative analysis.

Methods such as univariate and multivariate linear and logistic regression and chi-square analysis will be used. Subjects are expected to indirectly benefit from this study due to the general feeling of being rewarded for being able to help with research and for being able to advance the fields of social science and medicine. They are also likely to become more self-aware as they reflect upon their habits to answer some of the questions. There are no anticipated risks to the subjects other than the risk of confidentiality breaches for those from whom birthdates were collected. We have taken appropriate measures to mitigate this risk, as shown in the *Confidentiality* section. Respondents were not and will not be compensated in any way.

### Materials and Devices

The collection of the data was performed by using a survey instrument. After the data are collected, the generated spreadsheet will be analyzed by using Stata software.

### Confidentiality

No personally identifiable information (eg, the names of respondents, the address of houses, and any other contact information) was or will be collected through the use of a survey instrument. Birthdates were collected from some of the initial respondents, but these will be used exclusively to determine the participants’ ages. The birthdates will be destroyed thereafter.

### Consent

The terms of consent were explained to participants in the *Description* section of the survey. A large portion of the respondents belong to health care and allied fields (199/545, 36.5%); most of them are doctors and residents. Therefore, they already possess adequate knowledge about the purposes of the information collected for a research study. The rest of the respondents (346/545, 63.5%) were informed at the time of approach, and any pertaining questions were answered. Since filling out the form is optional and voluntary, consent is implied when a completed form is submitted. The exact paragraph that is presented in the form is shown in the *Questionnaire* section*.*

### Data Collection

The Google Forms software collects data automatically from the web-based survey instrument and converts responses from each corresponding question into a chart, table, or graph according to the most suitable method for pictorial representation. The software also generates a spreadsheet that contains all of the individual elements of information collected from a single survey (ie, in individual cells under columns and within rows). Individual entries from each survey form for further data analysis via varied methods are also available in this software.

### Questionnaire

The paragraph used for explaining consent is stated verbatim, as follows:

Title: Psychosocial and behavioral effects of Lockdown

Paragraph for consent: This form is for a research study to assess the psychosocial and behavioral effects of remote workers, students, and students in the transition to working due to graduation in the summer of 2020; in the lockdown or self-mandated quarantine due to COVID- 19 pandemic. It should take about 15-20 mins to fill out. We greatly appreciate you taking out the time to fill out this form and contributing to society and the field of science.

IMPORTANT: Your name is not required as a part of the survey and all the information you fill out will remain strictly confidential.

**Figure 1:**
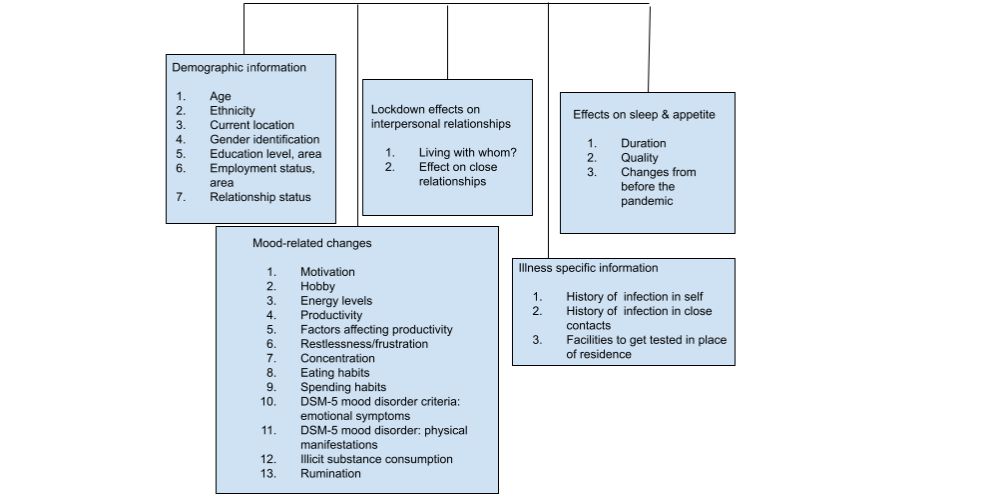
Questionnaire layout. *DSM-5*: *Diagnostic and Statistical Manual of Mental Disorders, 5th Edition*.

In Figure 1, the layout of the actual questionnaire is explained with the use of a simplified flowchart.

## Results

This study protocol was approved by the institutional review board at Vadilal Sarabhai General Hospital, Ahmedabad, Gujarat, India, on September 11, 2020. This study has received no monetary support. The collection of pilot data was started on June 10, 2020, and finished on September 15, 2020. The data of 550 participants were used for the preliminary data analysis and validation of the questionnaire. The data have been analyzed in depth with the use of Stata software, and we intend on publishing the data and the results of deeper statistical analyses once this protocol has been approved for publication.

## Discussion

A number of preliminary conclusions can be drawn by merely looking at the results data available. An important one is that 33.8% (184/545) of the total study population reported having high levels of sleep disturbances. Sleep disturbances have been linked to a decreased need for sleep resulting from less activity and increased amounts of psychological disturbances, such as anxiety and depression. Recent evidence from other studies in the literature has suggested that numerous psychological problems occur among the general public, health care workers, and patients with COVID-19 and has emphasized poor sleep quality, which is the most common psychological morbidity that has been observed during the COVID-19 pandemic [24]. We found that feelings of anxiety and agitation (183/545, 33.6%), restlessness (159/545, 29.2%), hopelessness and helplessness (122/545, 22.4%), and not being in control of anything (138/545, 25.3%) and difficulties with concentrating (194/545, 35.6%), etc, were reported by a high proportion of respondents. Irritability (169/545, 31%), the state of being easily fatigued (107/545, 19.6%), low or depressed mood (143/545, 26.2%), a lack of interest or diminished interest (114/545, 20.9%), the slowing down of thought processes (135/545, 24.8%), and excessive worry over physical appearance (145/545, 26.6%) were among the other psychological problems. Headaches (119/545, 21.8%), muscle tension (46/545, 8.4%), and heavy legs (58/545, 10.6%) and arms (20/545, 3.7%) were the least commonly reported psychological comorbidities.

Numerous other surveys have reported that a majority of households in India do not have access to high-quality foods such as vegetables and dairy products. An overwhelming majority of Bangladeshi people in low-income groups have reported that the pandemic has affected their livelihoods and have recorded high stress scores in addition to other negative psychosocial outcomes resulting from the worries regarding their livelihoods. Low socioeconomic classes have lower rates of financial literacy and lesser savings due to having the highest reliance on daily income [25].

COVID-19–related fear, moderate to severe depressive symptoms, and moderate to severe anxiety symptoms have been reported in other surveys and documented in the literature. The incidence of psychological disturbances has been reported and seen to be significantly higher in women. Respondents under the age of 30 years have reported lower levels of fear and depressive symptoms and have shown the least amount of social responsibility. Based on GLM, having a significant other with COVID-19, being on psychiatric medication, exhibiting safety and checking behaviors, and complying with guidelines are associated with higher levels of COVID-19–related fear. A linear regression analysis revealed that gender, age, and depressive and anxiety symptoms affect levels of COVID-19–related fear [26].

The results from our survey and all of the other evidence in the literature are proof that we need to direct our attention and health care resources toward the mental health of the population. Improving mental health can potentially to lead to increased motivation, willpower, and mental strength, which are some of the factors that can result in increased productivity and an overall better quality of life among the general population. Since our survey results are self-reported, they are limited by the lack of objectivity in the survey’s measures. However, the quality of life and psychological well-being of a person are personal issues that can vary from person to person. More detailed studies that are specifically tailored to an individual’s and target population’s mental well-being are required. Such studies would tremendously help with understanding the human psyche and aid researchers with making contributions to scientific literature.

## Abbreviations

DSM-5: *Diagnostic and Statistical Manual of Mental Disorders, 5th Edition*
